# Study on Pharmacokinetics and Metabolic Profiles of Novel Potential PLK-1 Inhibitors by UHPLC-MS/MS Combined with UHPLC-Q-Orbitrap/HRMS

**DOI:** 10.3390/molecules28062550

**Published:** 2023-03-10

**Authors:** Lin Wang, Hui Lei, Jing Lu, Wenyan Wang, Chunjiao Liu, Yunjie Wang, Yifei Yang, Jingwei Tian, Jianzhao Zhang

**Affiliations:** 1 School of Pharmacy, Key Laboratory of Molecular Pharmacology and Drug Evaluation, Ministry of Education, Collaborative Innovation Center of Advanced Drug Delivery System and Biotech Drugs in Universities of Shandong, Yantai University, Yantai 264005, China; 2 R & D Center, Luye Pharma Group Ltd., Yantai 264003, China; 3 College of Life Sciences, Yantai University, No. 30, Qingquan Road, Laishan District, Yantai 264005, China

**Keywords:** PLK-1, biological evaluation, pharmacokinetic, metabolic, UHPLC-MS/MS, UHPLC-Q-Orbitrap/HRMS

## Abstract

PLK-1 (Polo-like kinase-1) plays an essential role in cytokinesis, and its aberrant expression is considered to be keenly associated with a wide range of cancers. It has been selected as an appealing target and small-molecule inhibitors have been developed and studied in clinical trials. Unfortunately, most have been declared as failures due to the poor therapeutic response and off-target toxicity. In the present study, a novel potent PLK-1 inhibitor, compound **7a**, was designed and synthetized. ^1^H NMR, ^13^C NMR, ^19^F NMR and mass spectrum were comprehensively used for the compound characterization. The compound exhibited higher potency against PLK-1 kinase, HCT-116 and NCI-H2030 cell lines than the positive control. Molecular docking indicated that the binding mode that the ATP binding site of PLK-1 was occupied by the compound. Then, a UHPLC-MS/MS method was established and validated to explore the pharmacokinetic behavior of the drug candidate. The method had good selectivity, high sensitivity and wide linearity. The exposure increased linearly with the dose, but the oral bioavailability was not satisfactory enough. Then, the metabolism was studied using liver microsomes by UHPLC-Q-Orbitrap/HRMS. Our research first studied the pharmacokinetic metabolic characteristics of **7a** and may serve as a novel lead compound for the development of PLK-1 inhibitors.

## 1. Introduction

For many cancers, cell cycle dysregulation is a significant characteristic, while mitotic kinases play an essential role in cell cycle progression [1,2]. As a member of the serine/threonine protein kinase family, polo-like kinases (PLK) act as a regulatory protein, which is tightly associated with mitotic progression [3,4]. So far, five members of the PLK family have been identified in humans. Among them, PLK-1 is profoundly characterized and has been proven to be involved in checkpoint recovery, mitotic entry, centrosome maturation and bipolar spindle assembly [5,6,7]. The aberrant expression of PLK-1 was found in many malignant tumors and was strongly associated with poor prognosis [8,9,10]. Interestingly, PLK-1 seemed to be overexpressed only in dividing cancer cells for their endless proliferation and RNA silencing mediated deletion could arrest the cell cycle, trigger cancer cell apoptosis and inhibit tumor growth, but had no effect on normal cells [11,12]. By contrast, the function of other PLK family members was not so clear. Therefore, PLK-1 is considered a more promising and attractive target for anti-tumor drug design due to its better safety profile [5,13]. 

Small molecules selectively targeting PLK-1 have attracted much interest in the research community. Theoretically, both the N-terminal ATP-binding kinase domain (KD) and the C-terminal noncatalytic domains known as the polo-box domain (PBD) are druggable targets and corresponding inhibitors are under development [14,15]. However, only a few compounds have gained limited success in clinical trials [16]. GSK461364A is a thiophene amide derivative inhibitor but showed dose-limiting toxicity profiles, so the development was terminated [17]. Volasertib (BI 6727) was another ATP-competitive inhibitor with good oral availability but which failed in the Phase III study [18]. Onvansertib (NMS-P937), is another orally bioavailable selective inhibitor, which binds to the ATP-binding pocket of PLK-1, and blocked the phosphorylation of PLK-1 substrates. It has exhibited tumor growth suppression in hematologic, osteosarcoma, and colon adenocarcinoma cells [19,20,21]. Recent clinical trials have demonstrated its potential in treating head and neck squamous cell carcinoma [22], and acute myeloid leukemia [23]. However, patients only have partial responses when using monotherapy and serious adverse effects such as neutropenia and bone marrow suppression have been gradually emerged [23,24] and the latest progress have not yet been published. The development of PLK-1 inhibitors with high selectivity and efficacy remains a great challenge [25]. 

In addition, 2-(2-amino-pyrimidin-4-yl)-1,5,6,7-tetrahydro-pyrrolo[3,2-c] pyridin-4-one scaffold B was reported as a new chemical class of potent and selective PLK-1 inhibitor. Compound **25** demonstrated especially high activity and good selectivity versus a wide panel of kinases [26]. Recently, 1-methyl-2-(2-((5-(4-methylpiperazin-1-yl)-2-(trifluoromethoxy) phenyl) amino) pyrimidin-4-yl)-1,5-dihydro-4H-pyrrolo[3,2-c] pyridin-4-one (compound **7a**) was designed and synthetized to improve the efficacy. The PLK-1 activity and cytotoxicity of compound **7a** were a more than 4 times higher than compound **25**. As an ideal drug candidate requires appropriate pharmacokinetic characteristics in addition to excellent pharmacological activity, the pharmacokinetic of compound **7a** in rats was estimated by a validated UHPLC-MS/MS method. Furthermore, the metabolism was investigated using liver microsomes. This study can provide decision-making information for further research and the development of PLK-1 inhibitors.

## 2. Results and Discussion

### 2.1. Synthesis, Characterization and Biological Evaluation of Compound ***7a***

The target compound **7a** was synthesized according to the synthetic route displayed in Scheme 1 and was characterized and validated by ^1^H NMR,^13^C NMR, ^19^F NMR and mass spectrum (Supplementary Materials, Figures S1–S13).

**Figure 1:**
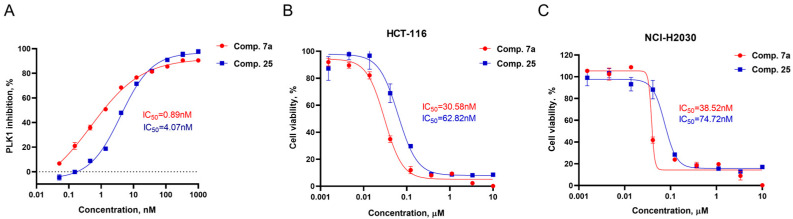
(**A**) The kinase activity of PLK-1, (**B**,**C**) Cell viability of **7a** and compound 25 in HCT-116 cell lines and NCI-H2030 cell lines.

Figure 1 A indicates the better PLK-1 kinase inhibitory activity of compound **7a** (IC_50_ = 0.89 nM) than **25** (IC_50_ = 4.07 nM). Consistent with the kinase activity, the treatment with **7a** produced a more significant anti-proliferative effect in HCT-116 (human colon cancer cell, Figure 1B) or NCI-H2030 (human non-small cell lung cancer cell, Figure 1C). 

### 2.2. UHPLC-MS/MS Method Development and Validation

**Figure 2:**
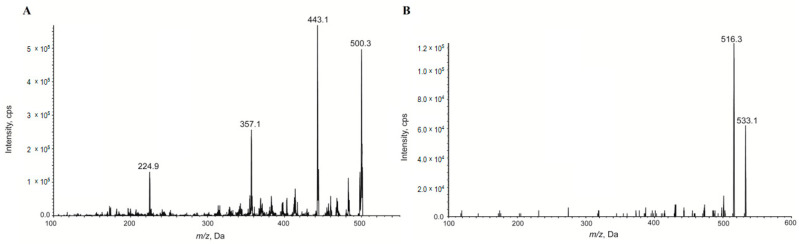
The MS/MS spectra of (**A**) **7a** and (**B**) onvansertib (IS) in positive mode.

A sensitive and rapid method was first developed by UHPLC-MS/MS first. The method was validated based on the Guidance for Industry on Bioanalytical Method Validation (FDA, 2018). In brief, precursor and fragment ions of **7a** and onvansertib (internal standard, IS) were acquired in the positive ionization mode (Figure 2).

**Figure 3:**
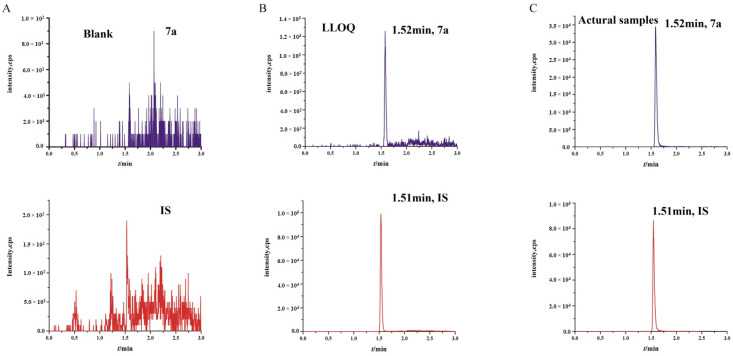
The representative chromatograms of **7a** and IS in (**A**) blank rat plasma, (**B**) blank plasma spiked with 1 nmol/L (0.5 ng/mL) of **7a** and 40 nmol/L (21.3 ng/mL) of IS, (**C**) plasma samples at 2 h after a single oral administration of compound **7a** (30 mg/kg).

The MRM ion pairs were 500.3/443.1 and 533.1/516.3 for **7a** and IS, respectively. For the higher intensity, 0.1% formic acid was added, and gradient elution was adopted for better chromatographic separation. The representative chromatograms are shown in Figure 3, and the retention times for **7a** and IS were 1.52 min and 1.51 min, respectively. No blatant interference existed.

**Figure 4:**
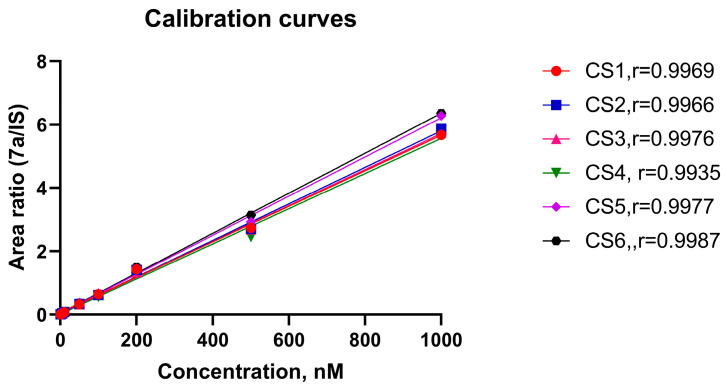
Calibration curves of compound **7a** over the concentration range of 1–1000 nmol/L (0.5–500 ng/mL). Red solid dot, blue squire, red regular triangle, green inverted triangle, purple parallelogram, and dark blue solid dot represent CS1 to CS6, respectively.

The calibration range of 1–1000 nmol/L (0.5–500 ng/mL) was determined according to the results of the pre-experiment of pharmacokinetics in vivo. Over the concentration range of 1–1000 nmol/L (0.5–500 ng/mL), the calibration curves showed good linearity (Figure 4). The lowest non-zero standard on the calibration curve defined LLOQ, and the signal to noise ratio was required to be greater than 10:1. The analyte response at the LLOQ should be more five times than the analyte response of the zero calibrator. The relative standard deviation (RSD, %) and relative error (RE, %) of LLOQ should be within ±20%. The sensitivity, precision and accuracy are summarized in Table 1, and the RSD of the intra-day and inter-day precision did not exceed 11.1%. The accuracy of the intra-day precision ranged from 88.70% to 100.46%, and the inter-day precision ranged from 91.24% to 105.50%. 

The peak areas of the simulated sample and the precipitated supernatant spiked with the corresponding solution were compared to calculate the recovery. Meanwhile, the blank plasma was replaced by pure water, and the peak area was compared with that of the supernatant to evaluate the matrix effect. As shown in Table 2, the extraction recovery of **7a** ranged from 76.3% to 81.8%, with the RSD being 5.09%. The matrix effect of **7a** ranged from 51.6% to 60.0%, with the RSD being 7.88%, suggesting that the matrix had no significant effect on the determination accuracy of the method. 

Both the RE and RSD in the stability test were less than ±15% (Table 3), indicating samples were stable in the current analytical method. In conclusion, the method was reliable, sensitive, accurate and reproducible, and could meet the quantitative requirements for biological samples.

### 2.3. Pharmacokinetic Study of Compound ***7a***

**Figure 5:**
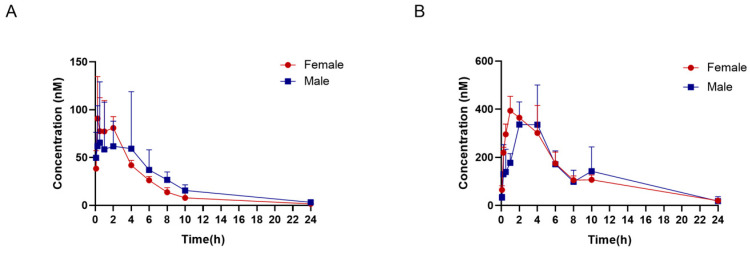
Mean concentration-time curves of **7a** in male and female rats after oral administration at a dose of 5 mg/kg (**A**) and 30 mg/kg (**B**). The data were presented as mean ± SD (n = 3).

The pharmacokinetic characteristics of **7a** were then evaluated successfully with the validated UHPLC-MS/MS method. Figure 5 displays the mean plasma concentrations-time profiles after the oral administrations of **7a**. Table 4 summarizes the corresponding pharmacokinetic parameters. There was almost no difference in C_max_ and AUC_0–24h_ between the male and female rats. The AUC_0–24h_ was about 517 and 3192 h × nmol/L at the doses of 5 and 30 mg/kg, respectively. The t_max_ was approximately 2 h, and t_1/2_ was 5 h. In the dose range of 5–30 mg/kg, the pharmacokinetics of **7a** were almost linear. The mean plasma concentration-time curve of **7a** after a single intravenous administration (1 mg/kg) and the major pharmacokinetic parameters can be found in Figure S14 and Table S1. The oral bioavailability (F) of **7a** was about 22%.

### 2.4. Metabolism of Compound ***7a***

**Figure 6:**
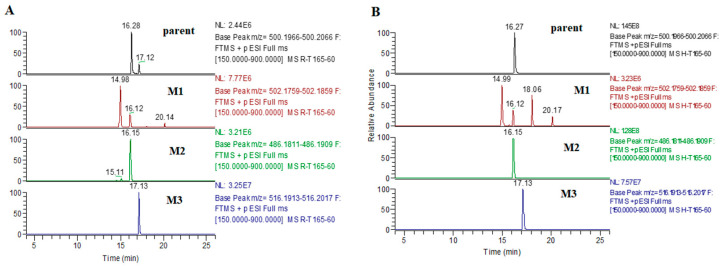
The extraction ion chromatogram of the compound **7a** and its metabolites in (**A**) rat liver microsomes and (**B**) human liver microsomes.

**Figure 7:**
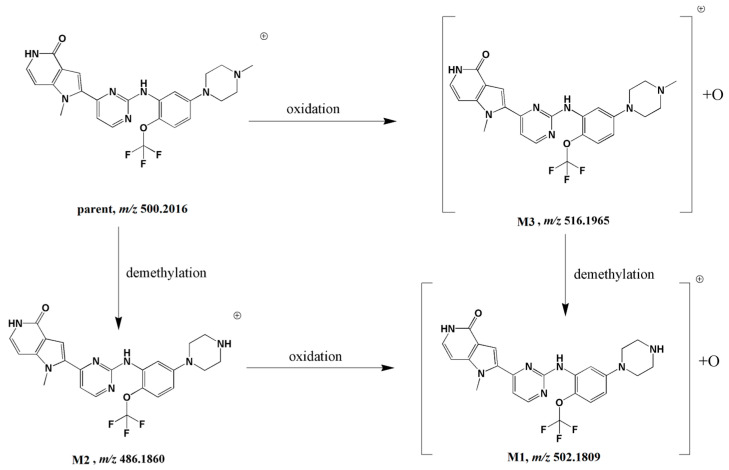
Speculated metabolic pathways of **7a**.

The metabolism of compound **7a** was explored using liver microsomes by UHPLC-Q-Orbitrap mass spectrometer. The extraction ion chromatogram is shown in Figure 6, and three primary metabolites were identified based on the accurate MS/MS spectra. The retention time, elemental composition, *m*/*z,* and area ratio are summarized in Table 5. Figure 7 describes the possible metabolic profiling. The mono-oxidation, and demethylation were the main metabolic pathways. The mono-oxidative metabolite, M3, was eluted after the parent, suggesting an N-oxide-type metabolite. In RLM, M3 (mono-oxidation) was the most abundant, based on the peak intensity. However, in HLM, the parent was stable, and the work might be of great significance for further research.

## 3. Materials and Methods

### 3.1. Materials

Onvansertib (purity 99.7%) was purchased from Med Chem Express (Monmouth Junction, NJ, USA). HPLC grade methanol and acetonitrile were obtained from Merck Life Science Co. Ltd. (Shanghai, China). Formic acid and phosphate-buffered saline were bought from Sigma Aldrich Trading Co., Ltd (Shanghai, China). Nicotinamide adenine dinucleotide phosphate (NADPH) was purchased from Roche Diagnostics GmbH (Mannheim, Germany). Ultrapure deionized water was obtained by a Milli-Q water system (Millipore, Burlington, MA, USA). The cell lines were purchased from the American Type Culture Collection (Manassas, VA, USA). PLK1 kinase (Batch NO. 05–157) was purchased from CarnaBio (Natick, MA, USA). The rat and human liver microsomes were purchased from Corning (Tewksbury, MA, USA). The HCT116 cell line was obtained from National Collection of Authenticated Cell Cultures (Shanghai, China). The NCI-H2030 cell line was obtained from the American type culture collection.

### 3.2. Synthesis of Compound ***7a***

Br_2_ (12.8 g, 80.3 mmol, 4.14 mL, 0.9 eq) was added to a mixture of compound **1** (15.0 g, 89.2 mmol, 1.00 eq) in HBr/AcOH (300 mL, 33% purity), drop-wise at 0 °C under N_2,_ and the mixture was stirred at 0 °C for 3 h. The three parallel reactions were combined to work up, and then the reaction was quenched with ice water (1500 mL) slowly and was extracted with EtOAc (300 mL × 4). The combined organic phase was washed with H_2_O (600 mL) and brine (200 mL), dried over anhydrous Na_2_SO_4_, filtered, and concentrated in vacuo to give compound **2** (64.0 g, crude) as a yellow solid. 

Compound 2a (17.3 g, 80.9 mmol, 1.00 eq) and NH_4_OAc (31.2 g, 405 mmol, 5.00 eq) were added into a mixture of compound **2** (20.0 g, 80.9 mmol, 1.00 eq) in EtOH (800 mL) at 10 °C under N_2_ and was stirred at 25 °C for 16 h. The three parallel reactions were combinedand the mixture was evaporated in vacuo. The crude was dissolved in EtOAc (800 mL) and filtered, and the filter cake was concentrated. The filtrate was washed with 1 N HCl (400 mL) and extracted with ethyl acetate (100 mL × 3). The organic phase was dried over Na_2_SO_4_ and evaporated in vacuo. The residue was purified by per-HPLC (neutral condition; column: Phenomenex Titank C18 Bulk 250 × 100 mm; mobile phase: [water (10 mM NH_4_HCO_3_)-ACN]; B%: 35%–70%, 20 min) to give compound **3** (21.6 g, 59.9 mmol, 24.7% yield) and compound 3A (39.8 g, 105 mmol, 43.2% yield) as a yellow solid.

Cs_2_CO_3_ (6.78 g, 20.8 mmol, 1.50 eq) and MeI (2.36 g, 16.7 mmol, 1.04 mL, 1.20 eq) at 10 °C were added to a mixture of compound **3** (5.00 g, 13.8 mmol, 1.00 eq) in DMF (100 mL) under N_2_ and the mixture was stirred at 33 °C for 12 h. The two parallel reactions were combined and the reaction mixture was filtered. The filter was quenched by NH_4_Cl (700 mL) slowly and then extracted with EtOAc (150 mL × 3). The combined organic phase was washed with brine (100 mL), dried over anhydrous Na_2_SO_4_, filtered and concentrated in vacuo to give compound **4** (10.3 g, crude) as a yellow solid.

m-CPBA (7.34 g, 34.1 mmol, 80% purity, 2.50 eq) was added to a mixture of compound **4** (5.10 g, 13.6 mmol, 1.00 eq) in DCM (200 mL), at 10 °C under N_2_, and the mixture was stirred at 10 °C for 12 h. The two parallel reactions were combined, and the reaction mixture was quenched with 10% aq. sodium metabisulfite (160 mL), and extracted with DCM (40 mL× 4). The organic layer was washed with aq. 10% sodium bicarbonate. The combined organic phase was dried over anhydrous Na_2_SO_4_, filtered, and concentrated in vacuo to give compound **5** (12.3 g, crude) as a yellow solid.

A solution of compound **5** (2.00 g, 4.92 mmol, 1.00 eq) in dioxane (45 mL) saturated with NH_3_·H_2_O (22.7 g, 195 mmol, 25 mL, 30% purity, 39.5 eq) was stirred under 15 Psi at 80 °C for 5 h in a 100 mL of a sealed tube. The three reactions were combined and the reaction mixture was concentrated in vacuo. Then the residue was dissolved with DCM (90 mL) and saturated Na_2_CO_3_ (60 mL). The aqueous phase was extracted with DCM (20 mL × 3). The combined organic phase was dried with anhydrous Na_2_SO_4_, filtered, and concentrated in a vacuum to give compound **6** (4.80 g, crude) as a yellow solid. 

Xantphos (599 mg, 1.04 mmol, 0.2 eq), Pd_2_(dba)_3_ (474 mg, 518 μmol, 0.1 eq) was added under N_2_ to a mixture of compound **6** (1.96 g, 5.70 mmol, 1.10 eq) and compound 6A (2.00 g, 5.18 mmol, 1.00 eq) in dioxane (80 mL), Cs_2_CO_3_ (3.38 g, 10.36 mmol, 2.00 eq). The mixture was stirred at 110 °C for 5 h and was concentrated in a vacuum. The residue was purified by column chromatography (SiO_2_, Petroleum ether/Ethyl acetate = 100/1 to 20/1) to give compound **7** (2.40 g, 2.19 mmol, 42.4% yield, 55% purity) as a brown solid. 

HCl/dioxane (4 M, 15 mL, 36.1 eq) was added to a mixture of compound **7** (1.00 g, 1.66 mmol, 1.00 eq) in THF (30 mL) under N_2_. The mixture was stirred at 25 °C for 16 h, and was concentrated at a reduced pressure to give a crude product. In total, 0.2 g crude product was purified by prep-HPLC (basic condition; column: Waters Xbridge Prep OBD C18 150 × 40 mm × 10 μm; mobile phase: [water (0.05% NH_3_·H_2_O +10 mM NH_4_HCO_3_)-ACN]; B%: 15%–70%, 8 min) to give compound **25** (12.0 mg) as a white solid. 

Dioxane (20 mL) was added DDQ (226 mg, 997 μmol, 2.50 eq) under N_2_ to a mixture of compound **25** (200 mg, 398 μmol, 1.00 eq). The mixture was stirred at 110 °C for 8 h, and was concentrated at a reduced pressure. The residue was poured into water (10 mL). The aqueous phase was extracted with ethyl acetate (2 mL × 4) and was concentrated in a vacuum. The residue was purified by prep-HPLC (HCl condition; column: Phenomenex Luna C_18_ 100 × 30 mm × 5 μm; mobile phase: [water (0.04% HCl)-ACN]; B%: 10%–40%, 10 min, and basic condition; column: Waters Xbridge BEH C_18_ 100 × 30 mm × 10 μm; mobile phase: [water (0.05% NH_3_·H_2_O + 10 mM NH_4_HCO_3_)-ACN]; B%: 5%–65%, 8 min) 2 times to give compound **7a** (5.00 mg, 10.01 μmol, 2.51% yield, 100% purity) as a light yellow solid. All compounds were confirmed by NMR or MS spectrum and the related information can be found in Supplemental Materials.

### 3.3. Kinase Inhibition Activity

The compounds were diluted at a 1:3 ratio on a 384-well plate with the highest concentration of 1 μM, and a total of 10 concentration points were tested. The mixture of PLK1 kinase and peptide substrate (5 μL) was added to each well. The plate was incubated at 23 °C for 15 min, and then 5 µL of ATP was added to initiate the reaction. After 15 min of incubation, PerkinElmer was added, and the system incubated for another 60 min at 23 °C. The results were tested by EnVision and analyzed using XLFIT5 software.

### 3.4. Cell Proliferation Inhibition Activity

HCT-116 or NCI-H2030 cells were seeded in 96-well plates at a density of 720/well and cultured overnight in a 5% CO_2_ incubator at 37 ± 1 °C. The compounds were diluted and added at a ratio of 1:3. After incubation for 72 h, ATPlite 1step Luminescence was added and incubated in the dark for 3 min, then was vortexed at 500 rpm for 2 min, and the luminous intensity was detected by the microplate reader and the cell inhibition was calculated as follows:

Inhibition (%) = 100 − (Lum compound − Lum medium)/(Lum control − Lum medium) × 100%

### 3.5. Method Development and Validation by UHPLC-MS/MS

Compound **7a** was quantitated by QTRAP 4500 tandem mass spectrometer coupled with electrospray ionization (SCIEX, Redwood City, CA, USA). Onvansertib was selected as the IS. The MS/MS spectrum were acquired in the positive ionization mode. Multiple reaction monitor (MRM) mode was selected for the quantification: 500.3/443.1 for **7a** and 533.1/516.3 for IS. The collision energies were 49 and 39 volts, respectively. The declustering potential was 120 volts, and the dwell time was 100 ms. Other mass spectrometry parameters were set as below: curtain gas, 10 psi; collision gas, “medium”; ion spray voltage, 5000 V; spray temperature, 500 °C; ion source gas 1, 50 psi; ion source gas 2, 50 psi. 

Exion UHPLC system (SCIEX, California, USA) with a YMC Triart Phenyl (50 mm × 2.1 mm, 3 μm; YMC CO., LTD, Kyoto, Japan) column was employed for the chromatographic separation. Ultrapure water containing 0.1% (*v*/*v*) formic acid (A) and methanol (B) were used as the mobile phase with a flow rate of 0.30 mL/min. The gradient elution procedure was used for better separation: 0 min, 20% B; 1 min, 95% B; 2 min, 95% B; 2.1 min, 20% B; 3 min, stop. The column temperature was 35 °C, and the auto-sampler temperature was 4 °C. The sample injection volume was 2 μL.

Furthermore, **7a** was dissolved and serially diluted with methanol to prepare the working solutions. Then, the working solutions were diluted ten times with blank rat plasma to prepare the calibration samples. The final concentrations for the calibration curves were 1, 5, 10, 50, 100, 200, 500, and 1000 nmol/L. The lower limit of quantitation (LLOQ) (1 nmol/L), and quality control (QC) (3, 300, and 750 nmol/L) were prepared in the same way. Then, 50 μL of simulated plasma or practical plasma was added with 150 μL of acetonitrile (40 nmol/L of onvansertib) and vortexed for 2 min. After centrifugation at 14,000 rpm for 5 min, the supernatant was transferred for UHPLC-MS/MS analysis. 

For selectivity, blank plasma samples from six different rats were collected. In total, 50 μL blank plasma was added with 150 μL acetonitrile as the double blank sample. Comparatively, 45 μL blank plasma was added with 5 μL LLOQ work solution, and 150 μL acetonitrile containing 40 nmol/L IS solution. The samples were extracted and analyzed similarly, and the chromatograms were compared. The response of compound **7a** in the blank plasma should not exceed 20% of the LLOQ at the same retention time. For IS, the response should not exceed 5%.

The calibration samples (1, 5, 10, 50, 100, 200, 500, 1000 nmol/L) and quality control samples (1, 3, 300, and 750 nmol/L) were prepared and analyzed. The calibration curve was built by linear regression with the peak area ratio (**7a**/IS) versus the nominal concentrations of **7a** in plasma. The weighting coefficient was set as 1/x^2^. The minimum concentration of the calibration curve was set as LLOQ. The correlation coefficient of the calibration curve should be over 0.99. The relative standard deviation (RSD, %) and the relative error (RE, %) of LLOQ should be within ±20%. The intra-day and inter-day precision and accuracy were evaluated by analyzing the quality control samples on three consecutive days. RE (%) and RSD (%) should not exceed ±15%.

For recovery, QC working solutions were mixed with plasma and precipitated with acetonitrile. Otherwise, blank plasma was precipitated by acetonitrile, and then after centrifugation at 14,000 rpm for 5 min, the corresponding QC working solutions were added to the supernatant. Meanwhile, QC working solutions were mixed with pure water rather than plasma to evaluate the matrix effect. The RSD of the recovery and matrix effect should not exceed 15%.

QC samples were stored under different conditions, such as room temperature for 4 h, frozen-thawed for 3 cycles, frozen at −20 °C for 7 days, or stored in an auto-sampler for 24 h. The sample concentrations were calculated by the following calibration curve. The RE (%) and RSD (%) should be less than ±15%.

### 3.6. Pharmacokinetic Study 

Sprague-Dawley rats (8 weeks old, 180–220 g) were provided by Pengyue Laboratory Animal Technology Co., Ltd (Jinan, Shandong, China). All rats were fed adaptively for 1 week (20 °C–24 °C, 50%–70% relative humidity, 12/12 h light/dark cycle). A standard diet and boiled water were provided. The rats fasted for over 12h with free access to water before the study. Furthermore, **7a** was dissolved DMSO (5%) and then diluted with saline. The rats were given **7a** by gavage at 5 mg/kg and 30 mg/kg. In addition, 200 μL of blood was collected into a 1.5 mL tube added with heparin at 5 min, 15 min, 30 min, 1 h, 2 h, 4 h, 6 h, 8 h, 10 h, and 24 h after administration. Plasma was acquired by centrifugation (8000 rpm, 5 min) and all samples were immediately stored at −20 °C immediately. The guidelines of the Animal Ethics Committee of Luye Pharma Group Ltd. were strictly enforced during the animal experiments to ensure animal welfare. 

### 3.7. The Metabolism of ***7a*** in Liver Microsomes

Liver microsomes were used to investigate the metabolism clearance of **7a**. In addition, 5 µL of rat liver microsome (20 mg/mL), 2 µL of **7a** or onvansertib (100 µM), and 173 µL of PBS buffer (0.1 M, pH 7.4) were added to 1.5 mL Eppendorf tubes. After pre-incubation for 10 min at 37 °C, the reaction was initiated by adding an NADPH-generating system (20 µL) into the microsomal suspension. A system without NADPH was set as a negative control. After incubations for 10 min, 30 min, or 60 min, 400 µL of ice acetonitrile was added to quench the reaction. The supernatants were injected into Q Exactive ™ combined quadrupole Orbitrap mass spectrometer (Thermo Scientific, Waltham, MA, USA) for metabolite profiling and identification. The concentration of the compound to be measured at 0 min was defined as 100%, and the concentration at other times was converted into a residual percentage. 

### 3.8. Data Analysis

The main pharmacokinetic parameters were calculated using Phoenix WinNonlin 7.0 via a non-compartmental model (Pharsight, Mountain View, CA). The absolute bioavailability of **7a** in rats was determined as (AUCi.g.(0 – 24 h) × dosei.g.)/(AUCi.v.(0 – 24 h) × dosei.g.) × 100%. Data were presented as mean ± SD.

## 4. Conclusions

PLK-1, one type of serine-threonine kinase, has proven to be important in cell proliferation. Its overexpression is positively correlated with tumorigenesis and poor prognosis. Over the years, several inhibitors have been developed but failed in subsequent clinical trials because of unexpected toxicity or a low therapeutical index in monotherapy. Therefore, in this study, a novel compound was designed and synthesized. The biological activity of compound **7a** was better than the positive control. The preclinical pharmacokinetic properties of **7a** in rats were reported first by a validated UHPLC-MS/MS method. Compound **7a** exhibited an approximately linear relationship between the exposure and the dose. The oral bioavailability was only about 22%. The metabolism of **7a** was analyzed in liver microsomes by UHPLC-Q-Orbitrap mass spectrometer. The result indicated the low exposure in rats was likely due to metabolism-mediated elimination. According to our study, **7a** needed to be modified to improve its pharmacokinetic characteristics for further development. 

## Figures and Tables

**Scheme 1:**
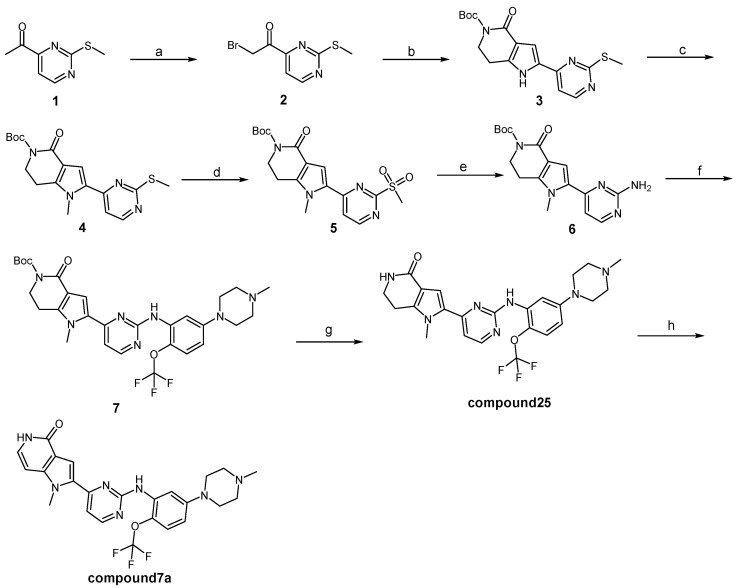
Synthetic route of compound **7a**. Reagents: (**a**) Br_2_, HBr, AcOH, 80 °C; (**b**) Tert-Butyl 2,4-Dioxopiperidine-1-Carboxylate, NH_4_OAc, EtOH, 10–25 °C; (**c**) Mel, Cs_2_CO_3_,DMF, 10–33 °C; (**d**) m-CPBA, DCM, 10 °C; (**e**) Dioxane, NH_3_·H_2_O, 10–33 °C; (**f**) 1-(3-iodo-4-(trifluoromethoxy)phenyl)-4-methylpiperazine, Cs_2_CO_3_, xantphos, Pd_2_(dba)_3_, Dioxane, 110 °C; (**g**) HCl/Dioxane, 10 °C; (**h**) DDQ, Dioxane, 110 °C.

**Table 1:** Intra-day and inter-day precision and accuracy of **7a** in rat plasma for three consecutive days.

	Nominal Concentration	Measured Concentration	Precision	Accuracy
	(nmol/L)	Mean ± SD (nmol/L)	RSD (%)	RE (%)
Intra-day (n = 6)	1	1.00 ± 0.07	6.46	0.46
	3	3.11 ± 0.22	7.19	3.67
	300	274 ± 10.7	3.91	−8.62
	750	665 ± 28.9	4.34	−11.3
Inter-day (n = 18)	1	1.06 ± 0.10	10.2	5.5
	3	2.77 ± 0.31	11.1	−7.67
	300	275 ± 13.5	4.91	−8.39
	750	684 ± 31.1	4.55	−8.76

**Table 2:** Recovery and matrix effects of **7a** and IS in rat plasma.

Analyte	Concentration	Recovery (%)		Matrix Effect (%)	
	(nmol/L)	Mean ± SD	RSD (%)	Mean ± SD	RSD (%)
**7a** (n = 6)	3.00	79.0 ± 3.54	5.09	51.6 ± 2.50	7.88
	300.00	76.3 ± 3.20		60.0 ± 1.98	
	750.00	81.8 ± 3.76		52.7 ± 1.77	
IS (n = 18)	40.00	107 ± 6.74	6.27	43.5 ± 0.83	3.52

**Table 3:** The stability of **7a** in rat plasma (n = 6).

Conditions	Nominal Concentrations (nM)	Measured Concentrations	RSD (%)	RE (%)
		(nM)		
Room temperature for 4 h	3	2.59 ± 0.17	6.47	−13.7
	300	261 ± 4.94	1.89	−13
	750	649 ± 17.9	2.76	−13.4
Freeze-thaw for 3 cycles	3	2.73 ± 0.21	7.56	−9.17
	300	260 ± 10.8	4.16	−13.4
	750	649 ± 24.2	3.74	−13.5
Autosampler (4 °C for 24 h)	3	3.05 ± 0.10	3.15	1.78
	300	280 ± 11.9	4.26	−6.67
	750	701 ± 46.5	6.63	−6.58
Freeze (−20 °C for 7 days)	3	2.55 ± 0.13	5.08	−14.9
	300	272 ± 9.67	3.55	−9.33
	750	685 ± 34.7	5.08	−8.71

**Table 4:** Main pharmacokinetics parameters of **7a** after oral administration at the doses of 5 and 30 mg/kg (mean ± SD, n = 3).

		T_1/2_ (h)	T_max_(h)	C_max_ (nmol/L)	AUC_0–24h_	F
					(h* nmol/L)	(%)
5 mg/kg	M	4.4 ± 0.9	3.0 ± 2.6	87 ± 45	565 ± 132	23.90%
	F	4.6 ± 1.4	0.8 ± 1.0	102 ± 25	469 ± 64	19.90%
	mean	4.5 ± 1.1	1.9 ± 2.1	95 ± 33	517 ± 106	21.90%
30 mg/kg	M	3.7 ± 1.3	2.0 ± 1.9	435 ± 81	3221 ± 1039	22.70%
	F	6.1 ± 0.9	2.3 ± 1.5	428 ± 90	3162 ± 1021	22.30%
	mean	5.1 ± 1.6	2.2 ± 1.6	432 ± 77	3192 ± 922	22.50%

**Table 5:** The metabolites of **7a** in liver microsomes and relative abundance.

Symbol	RT (min)	Elemental Composition	Observed *m*/*z*	Theoretical *m*/*z*	Error (ppm)	Mass Shift	Metabolic Pathway	RLM (%)	HLM (%)
parent	16.28	C_24_H_24_F_3_N_7_O_2_	500.2016	500.2022	−1.20	0	parent	5.34	43.73
M1	14.98	C_23_H_22_F_3_N_7_O_3_	502.1809	502.1814	−1.00	1.9793	+O-CH_2_	19.51	1.03
M2	16.15	C23H22F3N7O2	486.1860	486.1865	−1.03	−14.0156	-CH2	6.53	34.76
M3	17.13	C_24_H_24_F_3_N_7_O_3_	516.1965	516.1971	−1.16	15.9949	+O☐	68.62	20.48

## Data Availability

Data are contained within the article or Supplementary Materials.

## Supplementary Materials

The following supporting information can be downloaded at: https://www.mdpi.com/article/10.3390/molecules28062550/s1, Figure S1. 1H NMR (400MHz, DMSO-d6) spectrum of compound 2: δ 8.93 (d, J = 5.20 Hz, 1 H), 7.60–7.66 (m, 1 H), 5.02 (s, 2 H), 2.57–2.64 (m, 3 H); Figure S2. 1H NMR (400 MHz, CDCl3) spectrum of compound 3: δ 9.67 (br s, 1 H), 8.36 (d, J = 5.20 Hz, 1 H), 7.13–7.25 (m, 1 H), 7.06 (d, J = 5.20 Hz, 1 H), 4.06 (t, J = 6.40 Hz, 2 H), 2.91 (t, J = 6.40 Hz, 2 H), 2.50–2.57 (m, 3 H), 1.50 (s, 9 H); Figure S3. Mass spectrum of compound 4 (*m*/*z* 375.0); Figure S4. 1H NMR (400MHz, CDCl3) spectrum of compound 5: δ 8.64 (d, J = 5.60 Hz, 1 H), 7.57 (d, J = 5.60 Hz, 1 H), 7.33–7.41 (m, 1 H), 4.07 (t, J = 6.20 Hz, 2 H), 3.99 (s, 3 H), 3.28 (s, 3 H), 2.85–2.94 (m, 3 H), 1.50 (s, 9 H); Figure S5. Mass spectrum of compound 5 (*m*/*z* 306.9); Figure S6. Mass spectrum of compound 6 (*m*/*z* 344.2); Figure S7. Mass spectrum of compound 7 (*m*/*z* 602.3); Figure S8. 1H NMR (400 MHz, METHANOL-d4) spectrum of compound 25: δ 8.32 (d, J = 5.60 Hz, 1 H), 7.64 (d, J = 2.80 Hz, 1 H), 7.21–7.24 (m, 1 H), 7.19 (s, 1 H), 7.12 (d, J = 5.20 Hz, 1 H), 6.80 (dd, J = 9.20, 2.89 Hz, 1 H), 4.60 (s, 5 H), 3.87 (s, 3 H), 3.60 (t, J = 7.20 Hz, 2 H), 3.23–3.27 (m, 4 H), 2.95 (t, J = 7.20 Hz, 2 H), 2.62–2.66 (m, 4 H), 2.37 (s, 3 H); Figure S9. Mass spectrum of compound 25 (*m*/*z* 502.2); Figure S10. 1H NMR (400 MHz, METHANOL-d4) spectrum of compound **7a**: δ 8.42 (d, J = 5.20 Hz, 1 H) 7.69 (d, J = 2.80 Hz, 1 H), 7.42 (s, 1 H), 7.28 (d, J = 5.20 Hz, 1 H), 7.22–7.26 (m, 2 H), 6.81 (dd, J = 9.20, 3.01 Hz, 1 H), 6.74 (d, J = 7.20 Hz, 1 H), 4.02 (s, 3 H), 3.23–3.28 (m, 4 H), 2.61–2.65 (m, 4 H), 2.36 (s, 3 H); Figure S11. 13C NMR (400 MHz, DMSO) spectrum of compound **7a**: δ 160.8 ppm, 159.8 ppm, 158.8 ppm, 158.7 ppm, 150.6 ppm, 143.5 ppm, 134.9 ppm, 133.4 ppm, 133.2 ppm, 130.0ppm, 122.5 ppm, 115.3 ppm, 113.6 ppm, 112.2 ppm, 109.6 ppm, 93.76 ppm, 52.94 ppm, 48.40 ppm, 46.17 ppm, 33.83 ppm; Figure S12. 19F NMR (400 MHz, DMSO) of compound **7a**: δ −56.89 ppm; Figure S13. Mass spectrum of compound **7a** (*m*/*z* 500.2); Figure S14. Mean concentration-time curves of compound **7a** after intravenous administration in rats (1 mg/kg). Data were presented as mean ± SD, n = 3; Table S1. Main pharmacokinetics parameters of compound **7a** after intravenous administration (mean ± SD, n = 3).
