# Supplementary material for: Study on Pharmacokinetics and Metabolic Profiles of Novel Potential PLK-1 Inhibitors by UHPLC-MS/MS Combined with UHPLC-Q-Orbitrap/HRMS
Source: Molecules. 2023 Mar 10;28(6):2550. doi: 10.3390/molecules28062550 (PMC10053003; doi:10.3390/molecules28062550)
Supplement: Supplementary file 1 [file molecules-28-02550-s001.zip › molecules-2224007-supplementary.pdf]

Supplemental Materials

Study on pharmacokinetics and metabolic profiles of novel potential PLK-1 inhibitors by UHPLC-MS/MS combined with UHPLC-Q-Orbitrap/HRMS

Lin Wang, Hui Lei, Jing Lu, Xinfa Bai, Wenyan Wang, Chunjiao Liu, Yunjie Wang, Yifei Yang, Fangxia Zou, Liang Ye, Hongbo Wang, Jingwei Tian, Jianzhao Zhang

Results

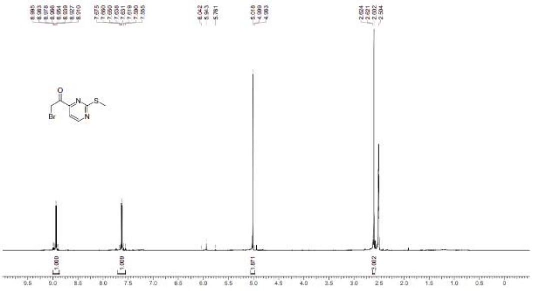

Figure S1. ^{1}H NMR (400MHz, DMSO-d6) spectrum of compound **2**: δ 8.93 (d, J = 5.20 Hz, 1 H), 7.60 - 7.66 (m, 1 H), 5.02 (s, 2 H), 2.57 - 2.64 (m, 3 H).

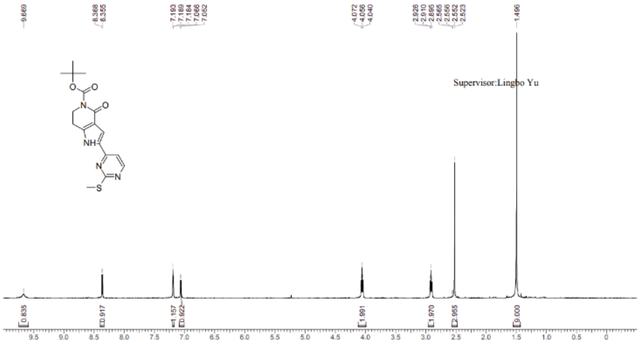

Figure S2. ^{1}H NMR (400MHz, $CDCl_3$) spectrum of compound **3**: δ 9.67 (br s, 1 H), 8.36 (d, J = 5.20 Hz, 1 H), 7.13 - 7.25 (m, 1 H), 7.06 (d, J = 5.20 Hz, 1 H), 4.06 (t, J = 6.40 Hz, 2 H), 2.91 (t, J = 6.40 Hz, 2 H), 2.50 - 2.57 (m, 3 H), 1.50 (s, 9 H).

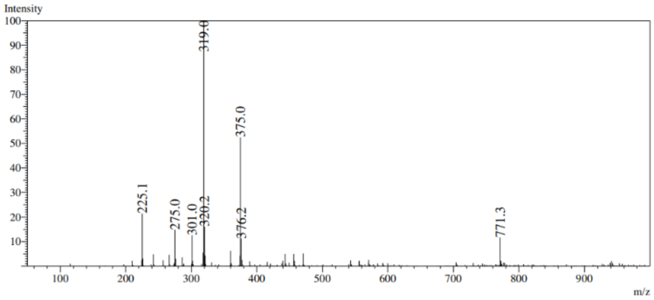

Figure **S3**. Mass spectrum of compound **4** (*m/z* 375.0).

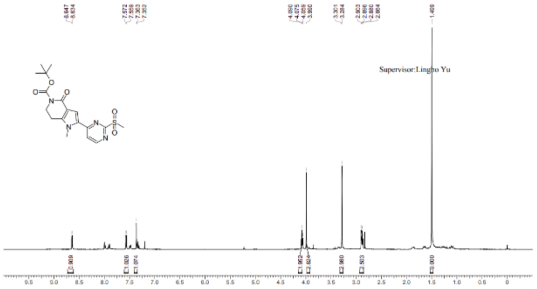

Figure S4. ^{1}H NMR (400MHz, $CDCl_3$) spectrum of compound **5**: δ 8.64 (d, J = 5.60 Hz, 1 H), 7.57 (d, J = 5.60 Hz, 1 H), 7.33 - 7.41 (m, 1 H), 4.07 (t, J = 6.20 Hz, 2 H), 3.99 (s, 3 H), 3.28 (s, 3 H), 2.85 - 2.94 (m, 3 H), 1.50 (s, 9 H).

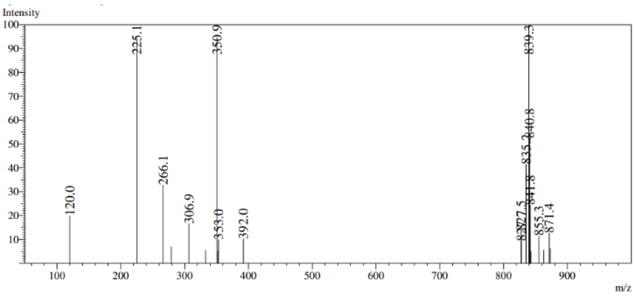

Figure **S5**. Mass spectrum of compound **5** (*m/z* 306.9).

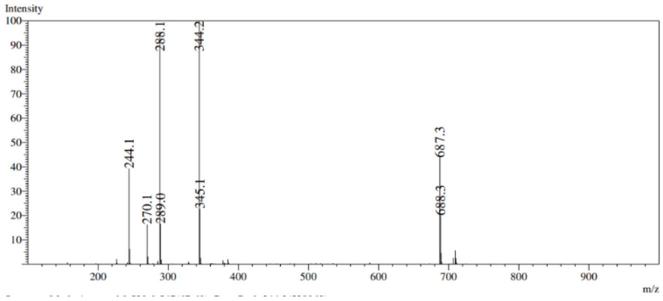

Figure **S6**. Mass spectrum of compound **6** (*m/z* 344.2).

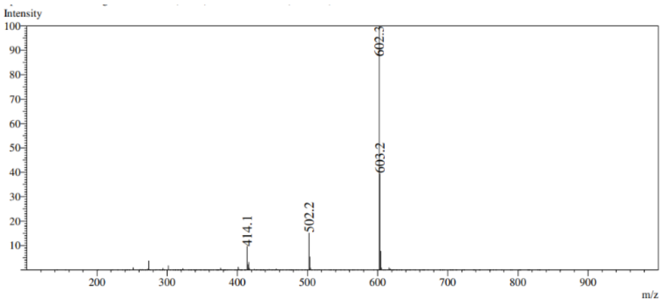

Figure **S7**. Mass spectrum of compound **7** (*m/z* 602.3).

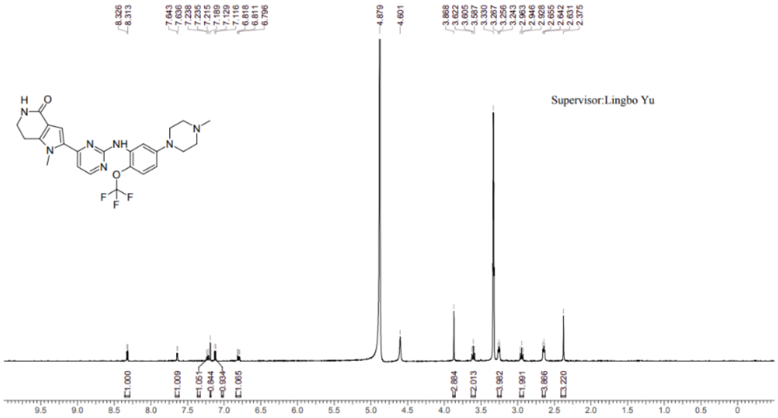

Figure S8. ^{1}H NMR (400MHz, METHANOL-d4) spectrum of compound **25**: δ 8.32 (d, J = 5.60 Hz, 1 H), 7.64 (d, J = 2.80 Hz, 1 H), 7.21 - 7.24 (m, 1 H), 7.19 (s, 1 H), 7.12 (d, J = 5.20 Hz, 1 H), 6.80 (dd, J = 9.20, 2.89 Hz, 1 H), 4.60 (s, 5 H), 3.87 (s, 3 H), 3.60 (t, J = 7.20 Hz, 2 H), 3.23 - 3.27 (m, 4 H), 2.95 (t, J = 7.20 Hz, 2 H), 2.62 - 2.66 (m, 4 H), 2.37 (s, 3 H).

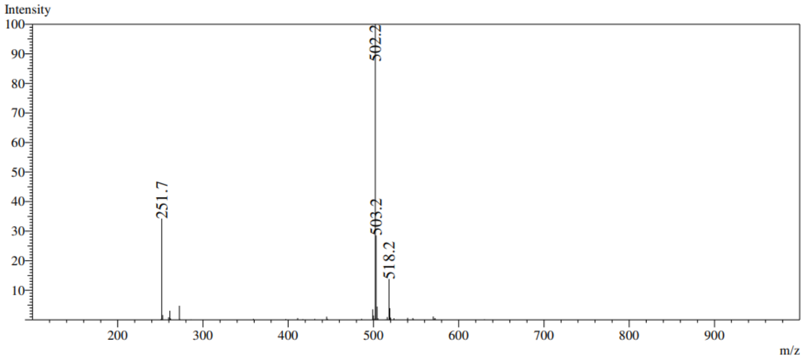

Figure S9. Mass spectrum of compound **25** (*m/z* 502.2).

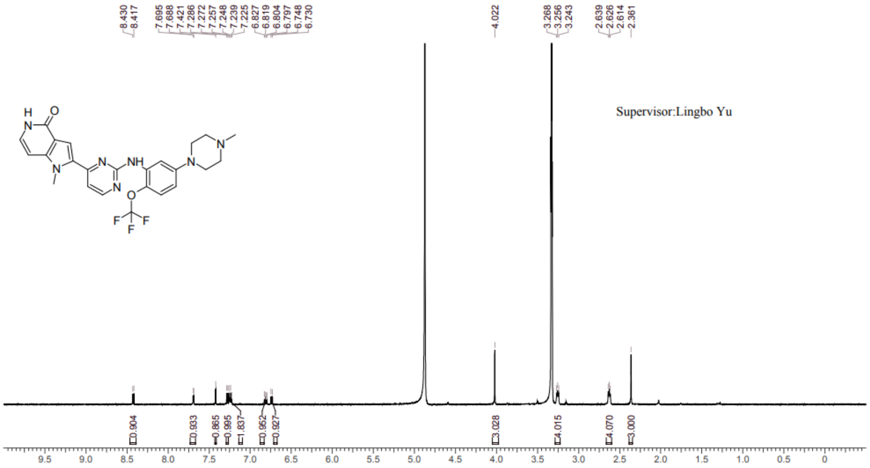

Figure S10. ^{1}H NMR (400MHz, METHANOL-d4) spectrum of compound **7a**: δ 8.42 (d, J = 5.20 Hz, 1 H) 7.69 (d, J = 2.80 Hz, 1 H), 7.42 (s, 1 H), 7.28 (d, J = 5.20 Hz, 1 H), 7.22 - 7.26 (m, 2 H), 6.81 (dd, J = 9.20, 3.01 Hz, 1 H), 6.74 (d, J = 7.20 Hz, 1 H), 4.02 (s, 3 H), 3.23 - 3.28 (m, 4 H), 2.61 - 2.65 (m, 4 H), 2.36 (s, 3 H).

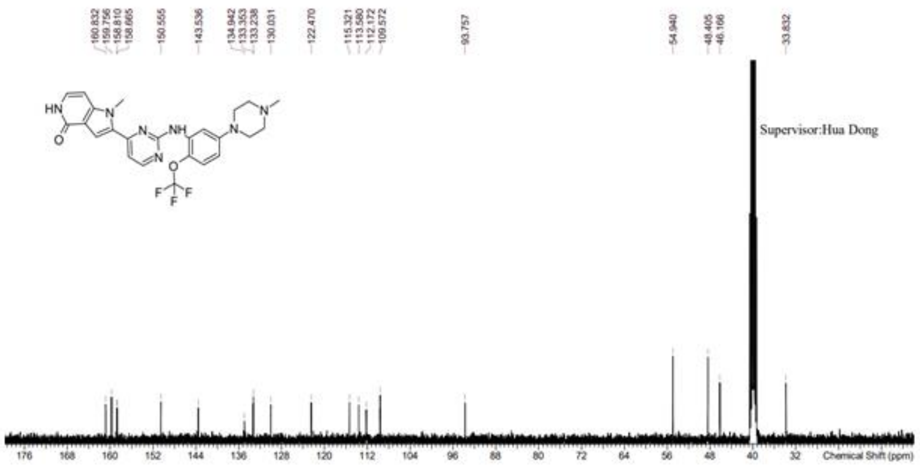

Figure S11. ^{13}C NMR (400MHz, DMSO) spectrum of compound **7a**: δ 160.8 ppm, 159.8 ppm, 158.8 ppm, 158.7 ppm, 150.6 ppm, 143.5 ppm, 134.9 ppm, 133.4 ppm, 133.2 ppm, 130.0ppm, 122.5 ppm, 115.3 ppm, 113.6 ppm, 112.2 ppm, 109.6 ppm, 93.76 ppm, 52.94 ppm, 48.40 ppm, 46.17 ppm, 33.83 ppm.

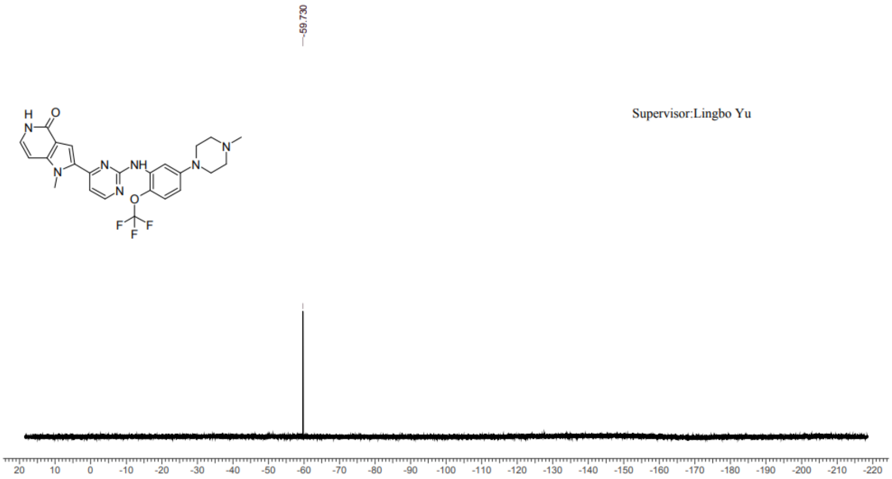

Figure S12. ^{19}F NMR (400 MHz, DMSO) of compound **7a**: δ -56.89 ppm.

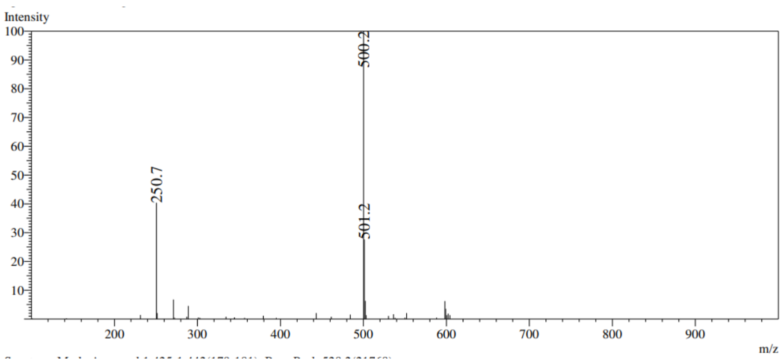

Figure S13. Mass spectrum of compound 7**a** (*m/z* 500.2).

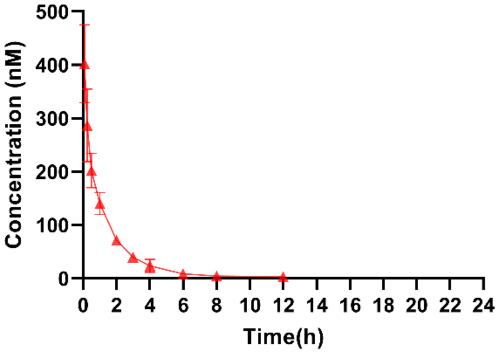

Figure S14. Mean concentration-time curves of compound **7a** after intravenous administration in rats (1 mg/kg). Data were presented as mean ± SD, n=3.

Table S1. Main pharmacokinetics parameters of compound **7a** after intravenous administration (mean ± SD, n=3).

	$T_{1/2}$ (h)	C_0 (nmol/L)	$AUC_{0\text{-}t}$ (h*nmol/L)	V_{ss} (L/kg)	Cl (L/h/kg)
i.v.	2.4±0.6	481±42	472±32	8.5±1.3	4.2±0.3
